# The ETS factor ESE3/EHF represses IL-6 preventing STAT3 activation and expansion of the prostate cancer stem-like compartment

**DOI:** 10.18632/oncotarget.12525

**Published:** 2016-10-08

**Authors:** Domenico Albino, Gianluca Civenni, Simona Rossi, Abhishek Mitra, Carlo V. Catapano, Giuseppina M. Carbone

**Affiliations:** ^1^ Tumor Biology and Experimental Therapeutics Program, Institute of Oncology Research (IOR), Bellinzona, Switzerland; ^2^ Oncology Institute of Southern Switzerland (IOSI), Bellinzona, Switzerland; ^3^ Department of Oncology, Faculty of Biology and Medicine, University of Lausanne, Lausanne, Switzerland

**Keywords:** ETS transcription factor, ESE3/EHF, IL-6, cancer stem cells, prostate cancer

## Abstract

Metastatic prostate cancer represents a yet unsolved clinical problem due to the high frequency of relapse and treatment resistance. Understanding the pathways that lead to prostate cancer progression is an important task to prevent this deadly disease. The ETS transcription factor ESE3/EHF has an important role in differentiation of human prostate epithelial cells. Loss of ESE3/EHF in prostate epithelial cells determines transformation, epithelial-to-mesenchymal transition (EMT) and acquisition of stem-like properties. In this study we identify IL-6 as a direct target of ESE3/EHF that is activated in prostate epithelial cells upon loss of ESE3/EHF. ESE3/EHF and IL-6 were significantly inversely correlated in prostate tumors. Chromatin immunoprecipitation confirmed binding of ESE3/EHF to a novel ETS binding site in the IL-6 gene promoter. Inhibition of IL-6 reverted transformation and stem-like phenotype in tumorigenic ESE3/EHF knockdown prostate epithelial cell models. Conversely, IL-6 stimulation induced malignant phenotypes, stem-like behavior and STAT3 activation. Increased level of IL-6 was observed in prostatospheres compared with adherent bulk cancer cells and this was associated with stronger activation of STAT3. Human prostate tumors with IL-6 elevation and loss of ESE3/EHF were associated with STAT3 activation and displayed upregulation of genes related to cell adhesion, cancer stem-like and metastatic spread. Pharmacological inhibition of IL-6/STAT3 activation by a JAK inhibitor restrained cancer stem cell growth *in vitro* and inhibited self-renewal *in vivo*. This study identifies a novel connection between the transcription factor ESE3/EHF and the IL-6/JAK/STAT3 pathway and suggests that targeting this axis might be preferentially beneficial in tumors with loss of ESE3/EHF.

## INTRODUCTION

Prostate cancer remain the most common malignancy and the second most frequent cause of cancer-related mortality in men in developed countries [1, 2]. Metastatic prostate cancer represents a yet unsolved clinical problem due to the high frequency of relapse and treatment resistance. Understanding the pathways that lead to prostate cancer progression in primary prostate tumors is an important task to prevent this deadly disease. Several studies have provided evidence of the presence of tumor-initiating stem-like cancer cells with high self-renewing properties in human cancers, including prostate cancer [3–5]. Cancer stem cells (CSCs) with acquired stem-like properties can originate from transformation of normal tissue/adult stem cells or from more differentiated progenitor cells [6, 7]. CSCs within the primary tumors are likely a major source of tumor heterogeneity, disease progression and treatment failure. The cancer stem cell model postulates a hierarchical organization of cells such that only a small subset is responsible for tumorigenesis in primary tumors. On the other hand, several factors contribute to the solid tumor heterogeneity including genetic mutations and epigenetic changes and the presence or absence of a cellular hierarchy. Thus, understanding the mechanisms controlling the expansion and maintenance of prostate CSCs could be an important step toward development of more effective CSC-directed strategies for treatment of prostate cancer.

ETS transcription factors are main elements in differentiation and developmental programs in many tissues. Expression of ETS factors is tightly regulated according to tissue-specific and time-dependent programs [8, 9]. Deregulated expression of ETS factors has oncogenic consequences on tissue developmental programs and is one of the most frequent findings in human tumors. About 50% of prostate cancers exhibit gene rearrangements and ectopic expression of ETS genes, like ERG and ETV1 [10–13]. ESE3/EHF is an ETS factor expressed in normal epithelial cells, including prostate epithelial cells [9]. Notably, ESE3/EHF ranks among most highly expressed transcription factors [14]. Previously we reported that ESE3/EHF is frequently downregulated in prostate tumors and that its loss is associated with robust inflammatory gene signatures [15, 16]. Furthermore, we showed that ESE3/EHF controls the differentiation program of prostate epithelial cells and its loss alters cell differentiation and conferred a CSC-like phenotype along with tumor-initiating and metastatic capability [17]. We found that ESE3/EHF controls a large network of targets transcriptionally, inducing genes related to epithelial cell differentiation and repressing genes connected with self-renewal and CSC phenotype [17]. Relevantly, we have recently shown that ESE3/EHF directly controls the level and activity of distinct components of the Lin28/let-7 axis, a key pathway involved in stem cell biology and expansion of cancer stem cell compartment [18].

IL-6 is a cytokine involved in many physiologic and pathophysiologic processes. IL-6 signaling leads to activation of JAK/STAT pathway and recent reports demonstrated that STAT3 activation occurrs frequently in metastatic prostate cancer [19]. While the oncogenic role of IL-6 has been widely investigated, little is known about factors regulating IL-6, particularly at the transcriptional level. Understanding the factors regulating IL-6 expression might be relevant for novel approach targeting its activation. In these studies we identify IL-6 as a novel direct target of ESE3/EHF. These data establish that repression of IL-6 is an important mechanism by which ESE3/EHF restrains stemness and tumor progression. This opens new perspective to target aggressive tumors with IL-6 elevation and loss of ESE3/EHF.

## RESULTS

### IL-6 is inversely correlated to ESE3/EHF in prostate cells and tumors

**Figure 1:**
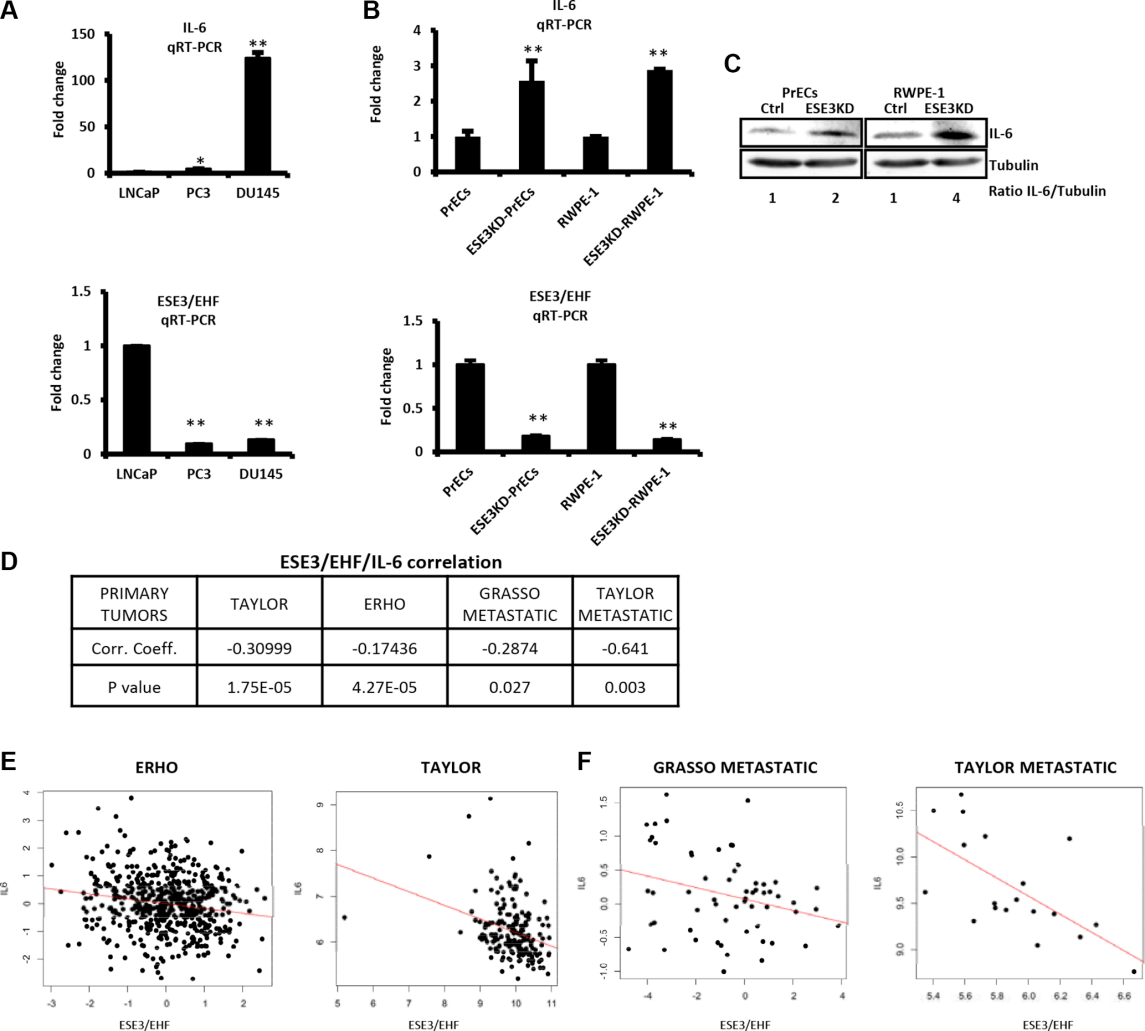
ESE3/EHF and IL-6 expression are inversely correlated (**A**) IL-6 (top) and ESE3/EHF (bottom) mRNA levels evaluated by qRT-PCR in indicated PCa cell lines. β-actin was used as reference for loading control. Data are presented as fold change relative to LNCaP cells. (**B**) IL-6 (top) and ESE3/EHF (bottom) mRNA levels evaluated by qRT-PCR in ESE3KD cell line models. β-actin was used as reference for loading control. Data are presented as fold change relative to PrECs and RWPE-1 cells. (**C**) Immunoblots of IL-6 in indicated cell lines. IL-6/Tubulin ratio determined by band intensity is reported. (**D**) ESE3/IL-6 correlation analysis. Table shows correlation coefficient and *p* value in indicated datasets. (**E**–**F**). Pearson distribution plots showing significant inverse correlation between IL-6 and ESE3/EHF in human primary (E) and metastatic prostate tumors (F). *P* values were determined using *t-test*. *P < 0.05; **P < 0.01. Data are representative of three independent experiments with at least three replicates per experiment.

In an effort to understand the role of ESE3/EHF in restraining stem-like phenotypes, we observed that in a panel of human prostate cancer cells IL-6 level was inversely correlated to ESE3/EHF and increased gradually from less aggressive, androgen-dependent and ESE3/EHF positive LNCaP to the more aggressive androgen-independent and ESE3/EHF negative DU145 cells (Figure 1A upper and lower panels). Moreover, IL-6 was significantly elevated in prostate epithelial cells with stable ESE3/EHF knockdown at the mRNA and protein level suggesting that ESE3/EHF could maintain IL-6 repressed in normal epithelial cells (Figure 1B and 1C). In keeping with this hypothesis, IL-6 level was significantly elevated in ESE3_low_ tumors (*p* < 0.01) compared to normal prostate [16]. To determine whether the link between ESE3/EHF and IL-6 observed in our cell line models was seen also in clinical samples, we analyzed gene expression data from two large (*n* = 545 and *n* = 131, respectively) human prostate cancer patients datasets [20, 21]. We found a significant inverse correlation between ESE3/EHF and IL-6 expression in human tumors (Figure 1D–1E). Notably, the inverse correlation between ESE3/EHF and IL-6 was also observed in metastatic prostate tumors (Figure 1F). Collectively, these data suggested that IL-6 could be a transcriptional target of ESE3/EHF.

### ESE3/EHF transcriptionally represses IL-6

**Figure 2:**
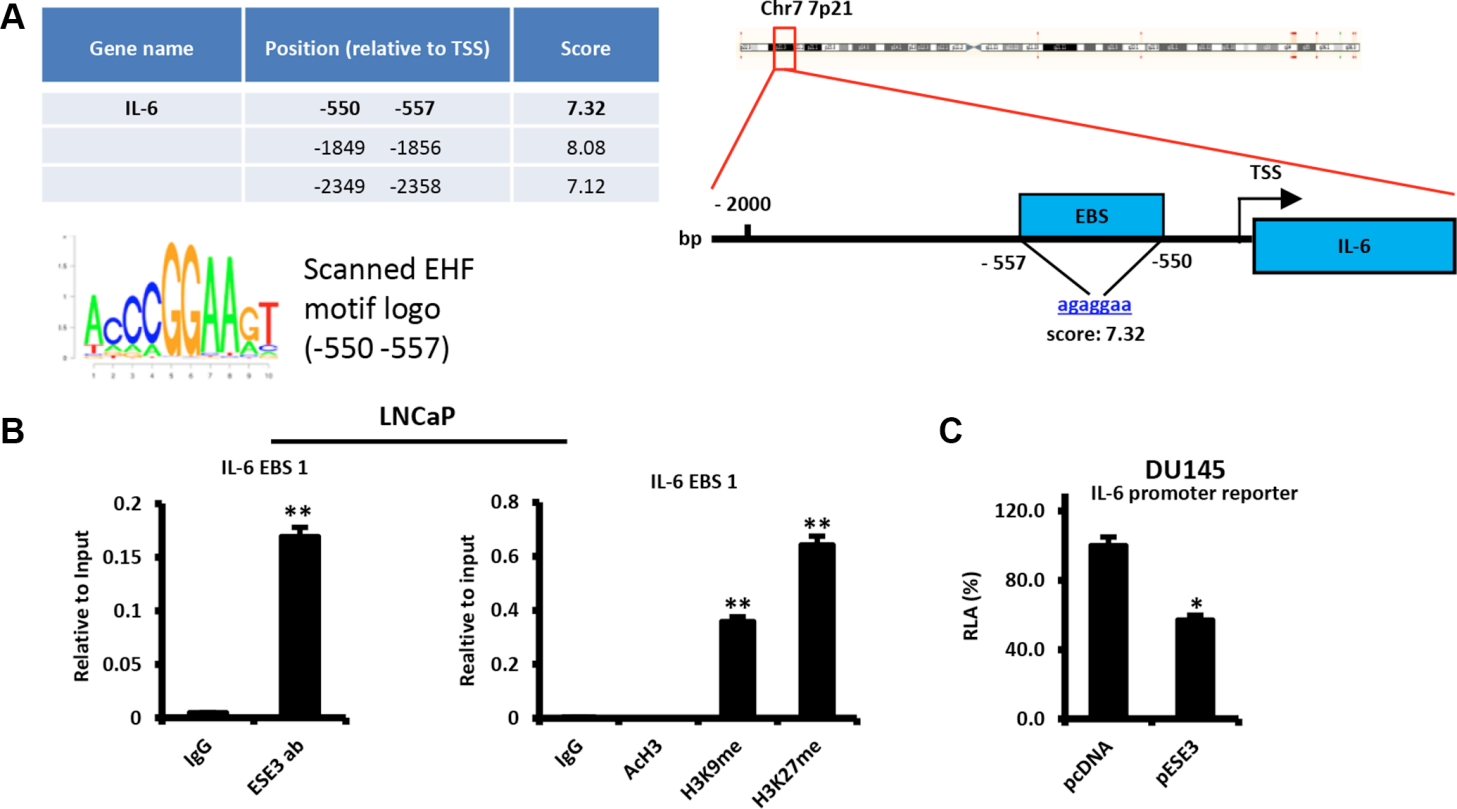
ESE3/EHF transcriptionally represses IL-6 (**A**) Predicted ETS binding sites (EBS) in the human IL-6 promoter (left). Position relative to the transcription start site (TSS) of the gene, sequence, corresponding scores (right) and EHF scanned motif logo (lower left). (**B**) Binding of ESE3/EHF to the IL-6 promoter (left panel) and chromatin marks (right panel) determined by chromatin immunoprecipitation (ChIP) in LNCaP cells. (**C**) Transcriptional activity of IL-6 promoter reporters in control (pcDNA) and stably ESE3/EHF expressing (pESE3) DU145 cells evaluated by dual luciferase assay. *P value*s were determined using *t-test*. *P < 0.05; **P < 0.01. Data are representative of three independent experiments.

To better define the relationship between ESE3/EHF and IL-6, we scanned the IL-6 gene promoter for ETS binding sites (EBS). Computational analysis showed multiple highly scored EBS corresponding to the consensus EHF motif in the promoter region (Figure 2A). To test whether ESE3/EHF bound to the promoter and controlled IL-6 transcription, we selected a high-confidence EBS which was also nearest (−550/−557 bp) to the transcription starting site (TSS) and performed chromatin immunoprecipitation (ChIP). We found that ESE3/EHF bound to the IL-6 promoter in LNCaP cells that express high level of ESE3/EHF (Figure 2B left panel). Consistent with a repressive function on the IL-6 promoter, we found enrichment of repressive (H3K9me and H3K27me) histone marks in LNCaP cells (Figure 2B right panel). The transcriptional effect of ESE3/EHF on IL-6 was further assessed by measuring IL-6 promoter activity in DU145 cells, which do not express endogenous ESE3/EHF. Activity of the IL-6 promoter reporter was significantly reduced by transient expression of ESE3/EHF in DU145 cells, consistent with transcriptional repression of the gene by ESE3/EHF (Figure 2C). Collectively these data support that ESE3/EHF directly control IL-6 transcription and maintains the gene under a repressive status.

### IL-6 is a mediator of prostate epithelial cell transformation and stem cell properties upon loss of ESE3/EHF

**Figure 3:**
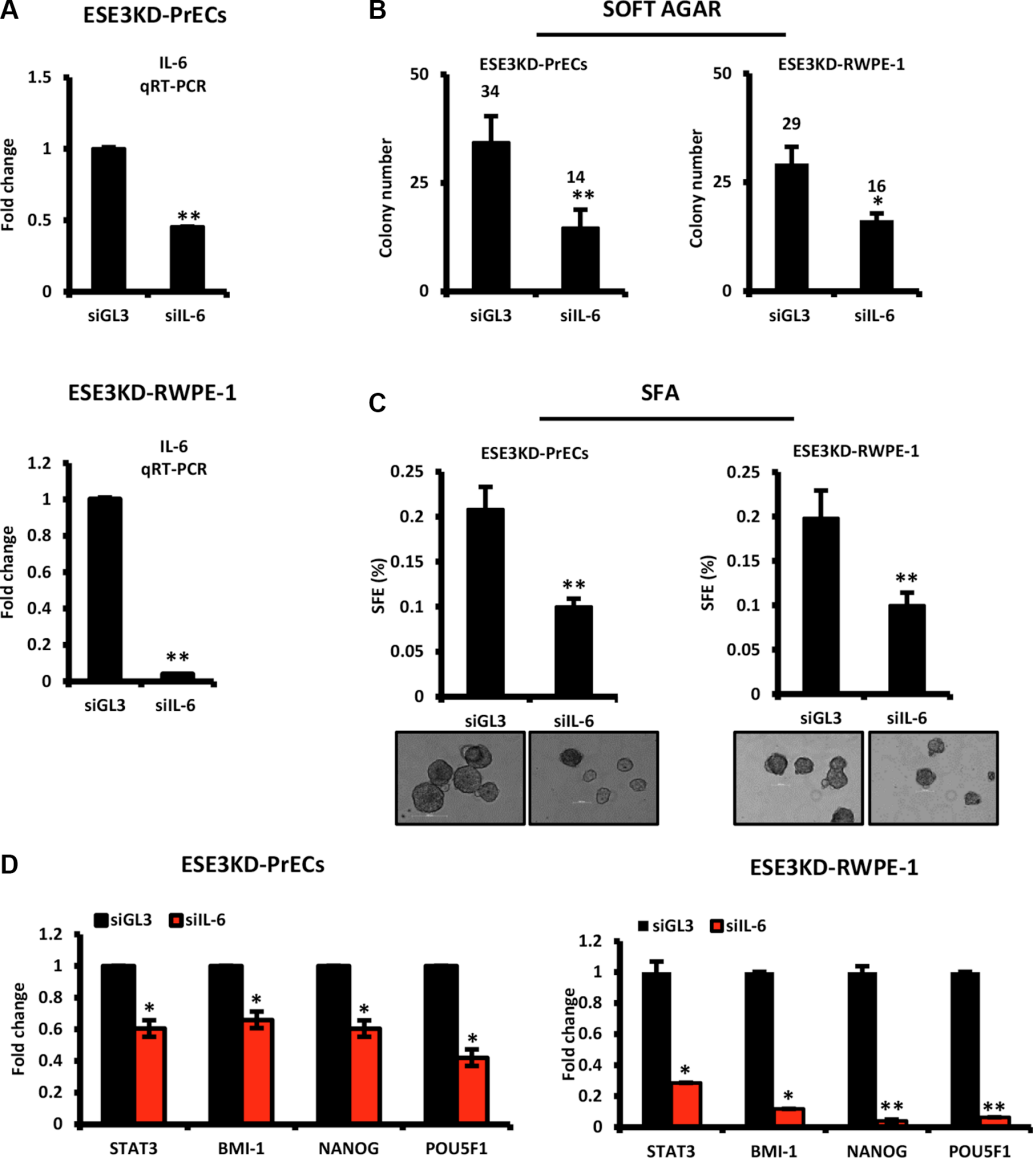
IL-6 is a mediator of prostate epithelial cell transformation and stem cell properties upon loss of ESE3/EHF (**A**) Knockdown efficiency of IL-6 evaluated by qRT-PCR following transfection with siRNAs targeting IL-6 in ESE3KD-PrECs (top) and RWPE-1 cells (bottom). (**B–C**) Colony formation (B) and sphere forming efficiency (C) in ESE3KD-PrECs and ESE3KD-RWPE-1 following transfection of IL-6 siRNA. (**D**) STAT3 and canonical CSC marker mRNA level evaluated by qRT-PCR in ESE3KD-PrECs (left) and ESE3KD-RWPE-1 (right) following transfection with siRNAs targeting IL-6. β-actin was used as reference gene. Data are presented as fold change relative to control siGL3. *P value*s were determined using *t-test*. *P < 0.05; **P < 0.01. Data are representative of three independent experiments.

To understand the contribution of IL-6 to the transformed phenotype observed in prostate epithelial cells upon loss of ESE3/EHF, we transiently knockdown IL-6 by siRNA in ESE3KD-PrECs and ESE3KD RWPE- 1 cells for 48 h and assessed the consequences on the cell phenotype. IL-6 knockdown was evaluated at mRNA level by qRT-PCR (Figure 3A). We observed a significant decrease in the colony number in soft agar in ESE3KD cells transfected with siRNA targeting IL-6 compared to control (siGL3) transfected cells (Figure 3B). Moreover, tumor sphere formation efficiency (SFE) was also significantly reduced in siIL-6 treated cells compared to control cells suggesting an impact on the cancer stem-like compartment (Figure 3C). To determine whether IL-6 ablation also reversed target gene activation observed in ESE3KD cells, we evaluated the expression of selected gene markers. We observed a significant reduction of the level of STAT3, BMI-1, NANOG and POU5F1 indicating that IL-6 activation contributes to the activation of these target genes upon loss of ESE3/EHF (Figure 3D). Collectively, these data indicate that knockdown of IL-6 in ESE3KD cells is sufficient to reverse the transforming and the stem-like phenotype acquired upon loss of ESE3/EHF. Thus, activation of the IL-6 pathway is an important mediator of the effects observed in ESE3KD cells and targeting IL-6 could be a useful strategy in the context of low ESE3/EHF expressing prostate tumors.

### IL-6 induces transformation and cancer stem-like phenotypes

**Figure 4:**
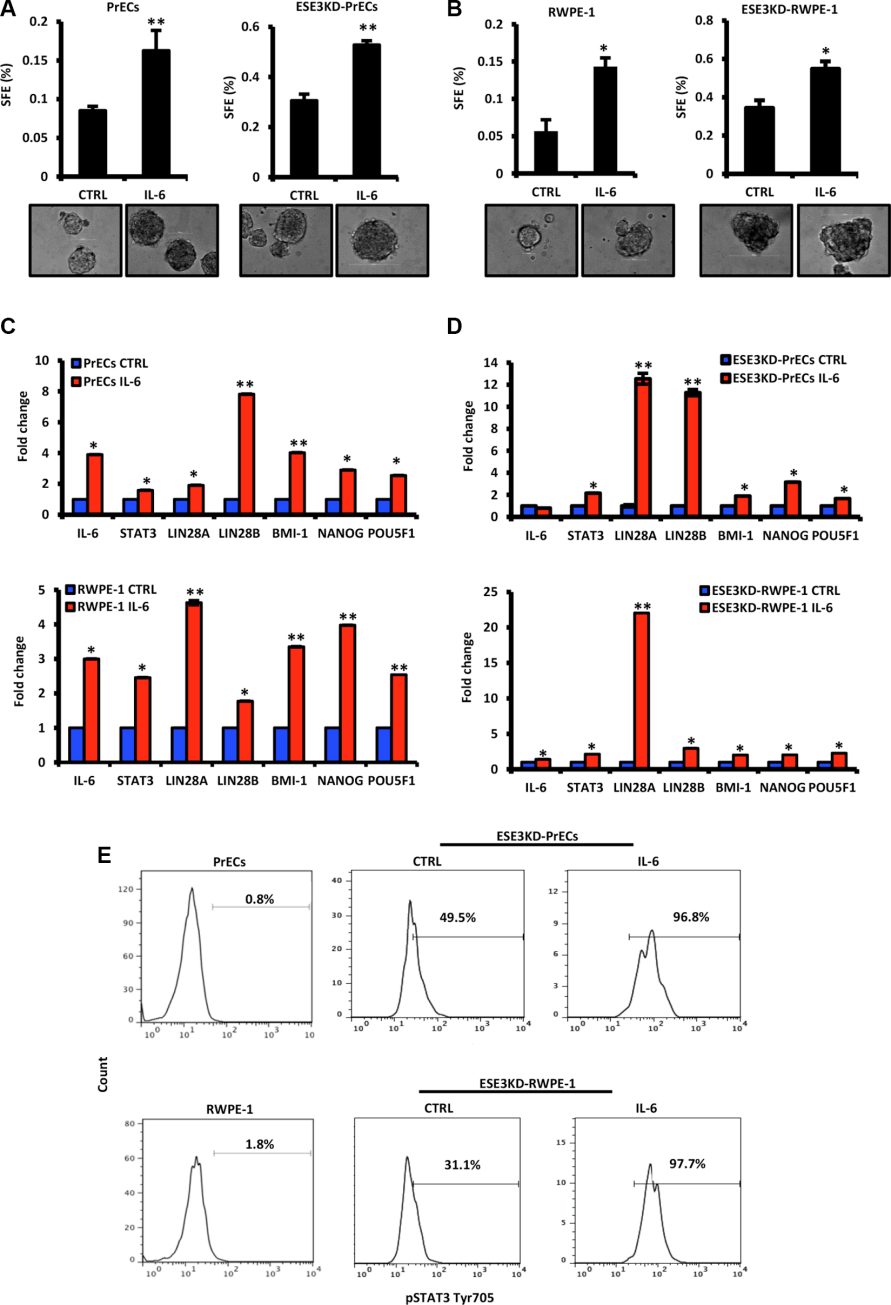
IL-6 induces transformation and cancer stem-like phenotypes in normal and more significantly in ESE3KD prostate epithelial cells (**A–B**) Sphere forming efficiency (SFE) in PrECs and ESE3KD-PrECs (A) and in RWPE-1 and ESE3KD-RWPE-1 (B) cells after IL-6 treatment *in vitro*. (**C–D**) mRNA levels of STAT3, IL-6 and indicated genes evaluated by qRT-PCR in PrECs and ESE3KD-PrECs (left) (C) and in RWPE-1 and ESE3KD-RWPE-1 cells (right) (D) following 4 hours of treatments *in vitro* with IL-6 (50 ng/ml) or DMSO as control (CTRL). Data are presented as fold change relative to control (CTRL) cells. (**E**) Flow cytometry analysis of STAT3-Tyr705 staining following IL-6 treatment of control PrECs and ESE3KD-PrECs (upper) and RWPE-1 and ESE3KD-RWPE-1 (lower) cells. Percentages of positive cells are shown. *P value*s were determined using *t-test*. *P < 0.05; **P < 0.01. Data are representative of three independent experiments.

To further understand the consequences of IL-6 upregulation, we exposed normal PrECs and RWPE-1 and corresponding ESE3KD cells to IL-6 stimulation. We observed that IL-6 treatment significantly increased tumor sphere formation in normal (PrECs) and ESE3KD prostate epithelial cells compared to vehicle treated cells (Figure 4A–4B). Consistently, canonical CSC markers like Lin28A, Lin28B, BMI-1, NANOG and POU5F1 were also significantly induced in both normal and ESE3KD cells compared to vehicle treated cells (Figure 4C–4D). IL-6 and STAT3 mRNA level were also induced after IL-6 treatment (Figure 4C–4D). Overall, the response to IL-6 was stronger in ESE3KD compared to the normal prostate epithelial cells. Flow cytometry analysis revealed that IL-6 stimulation induced a robust elevation of pSTATyr705 in ESE3KD-PrECs and ESE3KD-RWPE-1 in comparison to cells treated with vehicle (Figure 4E). Collectively these data support a role of IL-6 in expanding the prostate cancer stem-like phenotype and suggest that ESE3/EHF controls both basal and IL-6 induced STAT3 response. Low level of ESE3/EHF in the cell context and human tumors might render cells hypersensitive to IL-6 stimulation.

### Targeting IL-6/STAT3 activation by JAK2 inhibitors inhibits stemness and self–renewal properties in ESE3KD-PrECs *in vivo*

**Figure 5:**
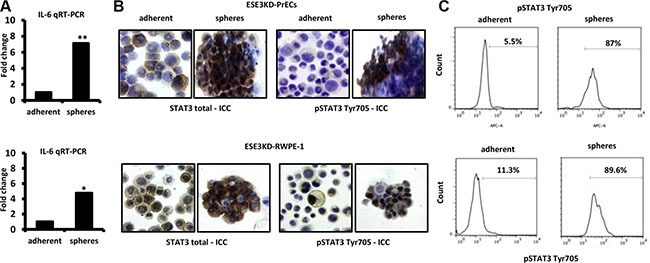
IL-6 elevation and STAT3 induction in cancer stem-like compartment of ESE3KD prostate epithelial cells (**A**) IL-6 mRNA level evaluated by qRT-PCR in adherent and prostatosphere cells derived from ESE3KD-PrECs (top) and ESE3KD-RWPE-1 (bottom) cells. Data are presented as fold change relative to the corresponding adherent cells. (**B**) ICC staining for STAT3 total and pSTAT3-Tyr705 for adherent cells and prostatospheres derived from ESE3KD-PrECs (upper) and ESE3KD-RWPE-1 (lower) cultures. (**C**) Flow cytometry analysis of STAT3-Tyr705 staining for indicated cells. Percentages of positive cells are shown.

IL-6 could play a role in transforming prostate epithelial cells by activating the JAK/STAT3 pathway. Consistent with this hypothesis, the level of IL-6 was significantly enriched in the CSC compartment in ESE3KD-PrECs in comparison to the adherent counterpart (Figure 5A). Intriguingly, we also found that pSTAT3 Tyr705 was increased in ESE3KD prostatospheres compared to adherent counterpart cells by ICC staining (Figure 5B) and by flow cytometry (Figure 5C) in ESE3KD-PrECs and RWPE-1 cells.

**Figure 6:**
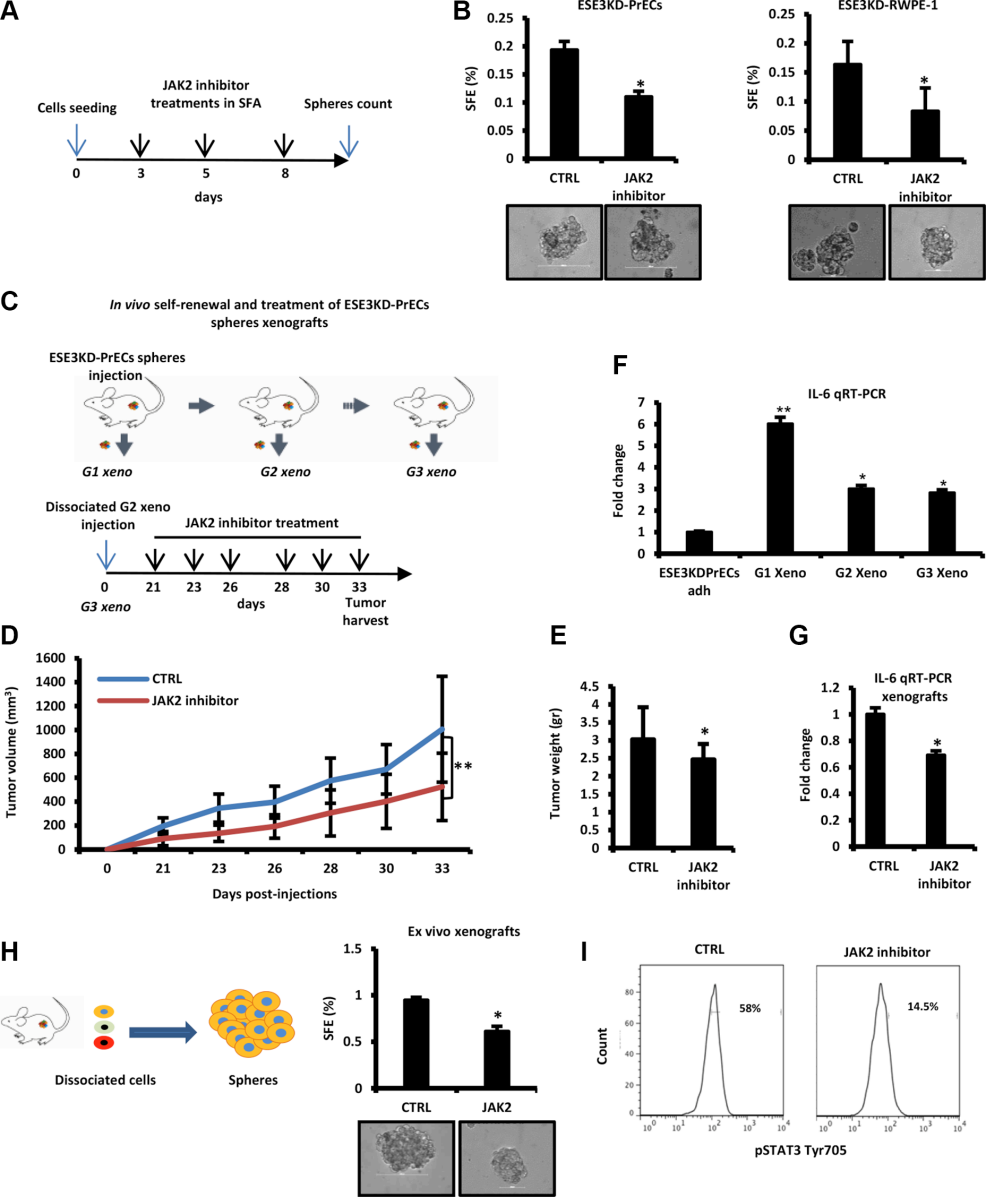
Targeting IL-6/STAT3 activation by JAK2 inhibitors reverses stemness and self–renewal properties *in vivo* (**A**) Experimental plan of JAK2 inhibitor (NVP) treatment of prostatospheres derived from ESE3KD-PrECs cells during SFA. (**B**) SFE of ESE3KD-PrECs and ESE3KD-RWPE-1 cells upon treatment described in A. (**C**) Experimental plan of the treatment with JAK2 inhibitor *in vivo*. Prostatospheres obtained from SFA were dissociated and injected subcutaneously in NSG mice (*n* = 4/group). Tumors formed by ESE3KD-PrECs prostatospheres cells (G1 xeno) were dissociated and re-implanted (2 × 10^5^ cells/site) in NSG mice (*n* = 4/group) for two consecutive generations (G2 xeno and G3 xeno). Treatment was initiated 21 days post-engraftment of the G3 xeno and continued as indicated (lower panel). (**D–E**) Tumor growth determined by caliper (D) and tumor weight (E) of G3 xeno following treatment as described in C. (**F**) IL-6 evaluation by qRT-PCR in ESE3KD prostatospheres and G1-G3 xeno. (**G**) IL-6 evaluation by qRT-PCR in control (CTRL) and following treatment with JAK2 inhibitor. (**H**–**I**). SFE *ex vivo* (H) and flow cytometry analysis of STAT3-Tyr705 staining (I) following JAK2 treatment described in C. *P value*s were determined using *t-test*. *P < 0.05; **P < 0.01. Data are representative of three independent experiments with at least three replicates per experiment. Percentages of positive cells are shown.

Thus, targeting the JAK/STAT3 pathway could antagonize stemness and self-renewal phenotypes in these cells. To test this, we used NVP-BSK805, a potent and selective JAK2 inhibitor, to inhibit STAT3 phosphorylation at Tyr705. NVP-BSK805 treatment significantly reduced prostatosphere formation in adherent ESE3KD-PrECs and ESE3KD-RWPE-1 cells (Figure 6A and 6B). Next, we tested the ability of NVP-BSK805 to inhibit tumorigenicity and self-renewal of ESE3KD-PrECs prostatosphere-derived tumor xenografts (ESE3KD-PrECs-xeno) *in vivo*. ESE3KD-PrECs xenografts were grown for the first generation *in vivo*. Then, the tumors were explanted, dissociated and re-engrafted subcutaneously. Treatment was initiated 21 days after the engraftment and continued for 2 weeks (Figure 6C). Notably, *in vivo* treatments significantly reduced the growth rate and size of the prostatosphere-derived tumor xenografts (Figure 6D– 6E). Notably, the level of IL-6 was elevated in G1-G3 prostatosphere-derived tumor xenografts being higher than those observed in ESE3KD adherent cells in culture (Figure 6F). Importantly, IL-6 was significantly reduced by the treatment with the JAK2 inhibitor (Figure 6G). Furthermore, when we explanted the tumors and evaluated *ex vivo* the SFE we found that the prostatosphere forming ability was significantly compromised in cells derived from NVP-BSK805 treated xenografts compared to control xenografts, suggesting that the CSC-like compartment was affected by the treatment (Figure 6H). Consistent with a specific inhibition of the JAK-STAT3 pathway, flow cytometry analysis revealed significant reduction of pSTAT3 in tumor xenografts treated with NVP-BSK805 (Figure 6I). Collectively, these data suggest that the expansion of stem-like cancer cells in tumor xenografts from ESE3KD-PrECs was associated with elevation of IL-6 and consequent activation of JAK/STAT3 and that it could be efficiently targeted by JAK/STAT3 inhibitors.

### IL-6 elevation and loss of ESE3/EHF are associated with STAT3 activation and enrichment of aggressive features in human prostate tumors

**Figure 7:**
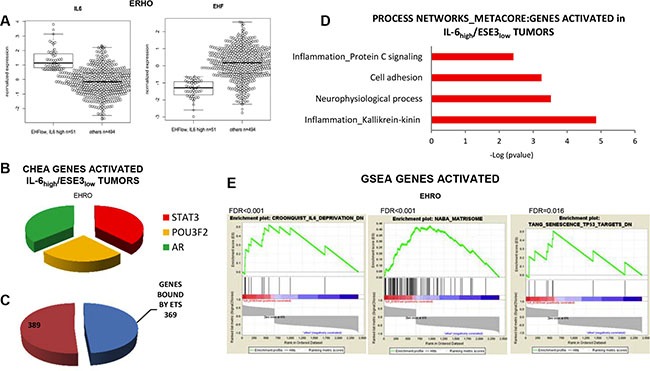
IL-6 elevation and loss of ESE3/EHF are associated with STAT3 activation and inflammatory features in human prostate tumors (**A**) Box–plots showing the level of IL-6 (left) and ESE3/EHF (right) in the selected tumor populations. (**B**) Transcription factor analysis of the top genes activated in IL-6_high_/ESE3_low_ compared to all the other tumors using CHEA by Enrich. (**C**) Number of ESE3/EHF putative binding sites among the top activated genes in the IL-6_high_/ESE3_low_ signature. (**D**) Functional annotation analysis by Metacore using the upregulated genes. (**E**) GSEA using indicated list of genes and comparing IL-6_high_/ESE3_low_ with all the other tumors in the EHRO dataset.

To further understand the clinical relevance of our data and verify the association between loss of ESE3/EHF, IL-6 upregulation and STAT3 activation with aggressive features in prostate tumors, we selected a group of tumors characterized by high level of IL-6 and low level of ESE3/EHF (IL-6_high_/ESE3_low_) (Figure 7A) in a large primary prostate tumor dataset [21]. By applying differential gene expression analysis we extracted a gene signature comparing the expression profile of the IL-6_high_/ESE3_low_ (*n* = 51) with the remaining tumors (*n* = 495). The IL-6_high_/ESE3_low_ tumors displayed a robust signature with several genes up and downregulated. We analyzed the top overexpressed genes (≥ 2 fold) in the IL-6_high_/ESE3_low_ signature by ChIP Enrichment Analysis (CHEA) and found a significant enrichment of STAT3 targets among other transcription factors (Figure 7B). Relevantly, the EBS sites were also significantly over-represented among the genes in the IL-6_high_/ESE3_low_ signature (Figure 7C) suggesting that the genes extracted could be direct targets of EHF. Functional annotation analysis of the genes activated in IL-6_high_/ESE3_low_ signature by Metacore supported enrichment of inflammatory genes and genes related to cell adhesion and metastatic spread (Figure 7D). To further understand the functional relevance of this signature we performed gene set enrichment analysis **(**GSEA) (Figure 7E). This analysis revealed that the IL-6_high_/ESE3_low_ tumors were significantly enriched of genes attenuated in IL-6 deprivation (Croonquist_IL-6_DN), underlying the accuracy of our approach in extracting relevant IL-6 targets in prostate tumors. Moreover, the IL- 6_high_/ESE3_low_ tumors were also enriched of an ensemble of genes encoding extracellular matrix and extracellular matrix-associated proteins (Naba_Matrisome), genes conferring migratory and metastatic properties. Intriguingly, there was also enrichment of genes acting as a barrier for senescence, a pathway opposing the induction of a cancer stem cell phenotype. Collectively, these findings supported the activation of the IL-6/STAT3 pathway and aggressive cancer stem-like phenotype in IL-6_high_/ESE3_low_ tumors and suggested that these tumors could be targeted by therapy opposing this pathway.

## DISCUSSION

**Figure 8:**
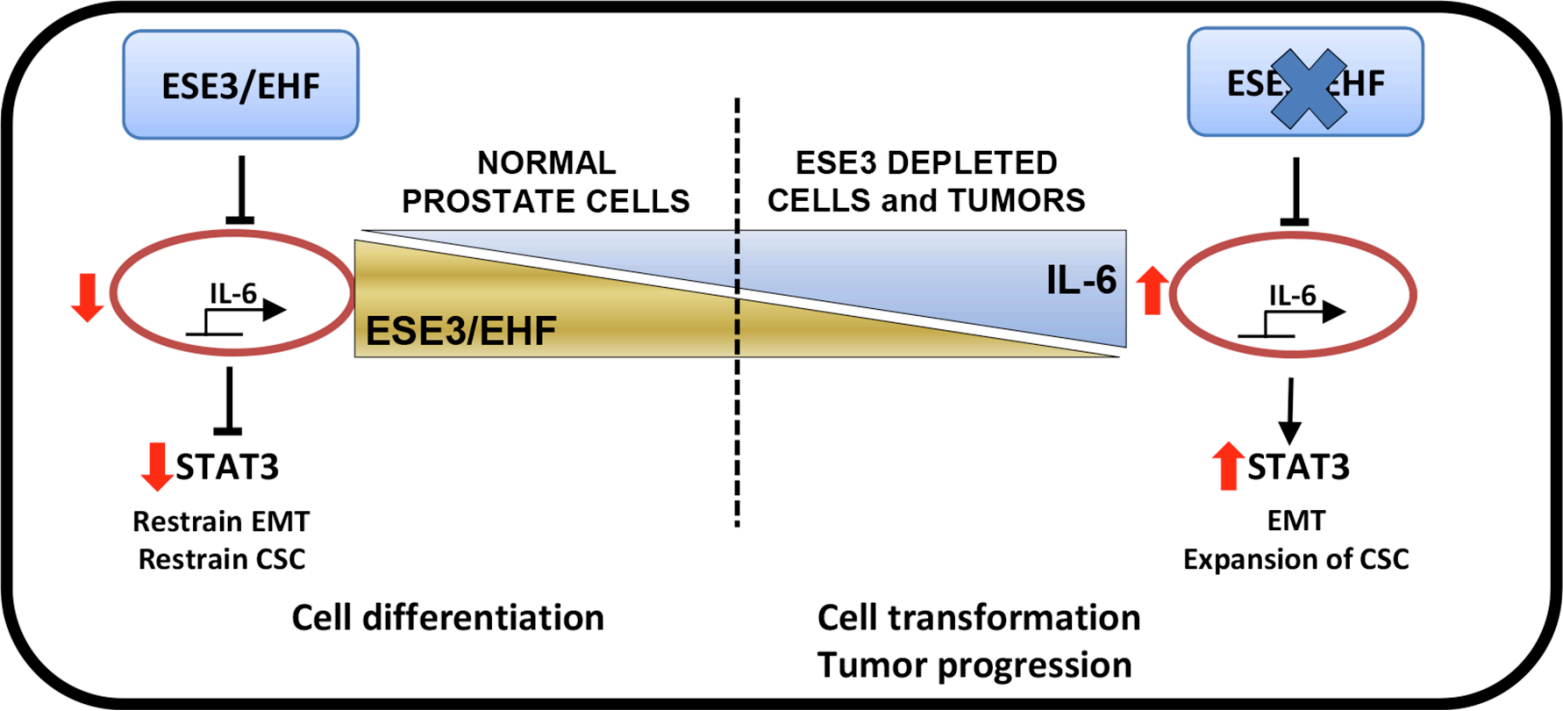
ESE3/EHF controls the activation status of IL-6 and JAK/STAT pathway in normal prostate Loss of ESE3/EHF leads to upregulation of IL-6 and activation of the JAK/STAT3 pathway with consequent induction of EMT and expansion of the cancer stem cell compartment.

In this study we report a link between the tumor suppressor ESE3/EHF and IL-6. Specifically, loss of ESE3/EHF leads to upregulation of IL-6 and activation of the JAK/STAT3 pathway with consequent induction of EMT and expansion of the cancer stem cell compartment (Figure 8).

The ETS factor ESE3/EHF is an endogenously expressed ETS factor whose level is higher in normal prostate and decreased in aggressive and metastatic tumors [15]. We recently reported a tumor suppressor role of ESE3/EHF in controlling differentiation and cancer stem-like phenotype in human prostate epithelial cells [17]. ESE3/EHF loss in prostate epithelial cells leads to cell transformation, EMT, acquisition of stem-like properties and broad reprogramming of the cell transcriptome. More recently, we have reported that ESE3/EHF controls the Lin28/let-7 axis acting as a critical barrier to malignant transformation and preventing cancer stem cell expansion [18]. In this study, in an effort to further understand the mechanisms by which ESE3/EHF controls cancer stem-like cells we uncovered that IL-6 is a key target repressed by ESE3/EHF. IL-6 is a cytokine involved in many pathophysiologic processes. IL-6 signals through a cell-surface type I cytokine receptor complex. IL-6 interaction with the receptor triggers its activation initiating a signal transduction cascade leading to activation of the JAK/STAT pathway. Several pieces of evidences have associated IL-6 with an aggressive prostate cancer phenotype and metastatic processes through the regulation of EMT and the homing of cancer cells to the bone [22]. Recent evidence indicates that IL-6 plays a major role in the transition from hormone-dependent to CRPC [22]. Moreover, IL-6 is upregulated in many epithelial cancers such as breast and prostate [23–25]. Collectively, the evidence reported so far points to an important role of IL-6 in transformation and a stem-like phenotype in tumors. Additionally, IL-6 is a key molecule in the dynamic equilibrium between CSCs and non cancer stem cells (NCSCs) via IL-6 secretion, and IL-6 can convert NCSCs to CSCs in breast and prostate cell lines as well as from cells derived from human breast tumors [26]. Importantly, IL-6 has been reported as a key mediator of an inflammatory positive feedback loop occurring from non-transformed to transformed breast epithelial cells and involving other key oncogenic proteins such as NF-kB, Lin28 and STAT3 [27].

Thus, a deep understanding of factors regulating IL-6 expression might be relevant for the development of novel therapeutic approaches targeting its activity [28, 29]. We have previously reported that IL-6 is induced in prostate cancer cells and that the ETS factor ESE1 was an important mediator enhancing the NFKb response [30].

In this study we identify IL-6 as a novel direct target of ESE3/EHF in prostate epithelial cells. Intriguingly, to our knowledge this is the first reported inhibitory transcription factor controlling IL-6.

IL-6 expression has been reported to be inhibited by wild type p53 and the retinoblastoma (RB) tumor suppressor gene [31]. However, it is not known if the effect is direct or mediated by the modulation of other transcription factors. Future studies should address the possibility of a crosstalk between ESE3, Rb and p53 in the control of IL-6 transcription.

We have recently reported that ESE3/EHF restrains stemness by repressing the LIN28/let7 axis [18]. The data presented here establish an additional mechanism by which ESE3/EHF restrains stemness and tumor progression through repression of IL-6. On the other hand, elevation of IL-6 by loss of ESE3/EHF might also impact the activation of Lin28/let7b axis. Accordingly, we found that IL-6 treatment promoted transformation and cancer stem-like features in normal prostate epithelial cells. In contrast, IL-6 inhibition by siRNA reverted these malignant phenotypes in cancer cells. We have previously reported that tumors with low levels of ESE3/EHF have aggressive clinical characteristics and are phenotypically associated with EMT and cancer stem cell features. The data reported here support the notion that IL-6 could contribute to the aggressive features of these tumors and particularly the cancer stem like compartment. Tumors with low levels of ESE3/EHF might be more prone to an aberrant response to IL-6 stimulation. Collectively, these data open new perspectives to specifically target this group of tumors with strategies reversing IL-6 activation. We report here that treatment with JAK-STAT inhibitors prevented self-renewal *in vivo* in prostate epithelial cells with ESE3/EHF knockdown. These data are clinically relevant and suggest the possibility of selective targeting of the CSC like compartment in ESE3_low_ tumors.

Although the use and benefit of JAK-STAT inhibitors has been amply reported, our data introduce a novel concept that targeting IL-6-JAK-STAT3 signaling could be more effective in tumors in which both ESE3/EHF and IL-6 are altered. Further studies are necessary to demonstrate this concept. IL-6 has been shown to have context-dependent pro- and anti-inflammatory properties and this might influence greatly its oncogenic capabilities in different contexts [29]. Interestingly, recent data from our laboratory indicate that there are additional molecular mechanisms linking to IL-6/STAT3 activation in the context of ESE3/EHF loss in addition to the one described here. Collectively, our data support the notion that activation of the IL-6/STAT3 and inflammatory-like signaling leads to deleterious effects in normal prostate epithelial cells and tumors, leading to expansion of the cancer stem-like cell compartment and self-renewal properties. Understanding these mechanisms will support the possibility of targeting these aggressive tumors within the scope of development of precision medicine approaches for the prostate cancer management and treatment.

## MATERIALS AND METHODS

### Cell lines, transfection and selection of cell clones

Immortalized human prostate epithelial cells (PrECs) [15] and RPWE-1 with stable knockdown of ESE3/EHF by shRNAs were established as previously described [16]. LNCaP, DU145 and PC3 were obtained from ATCC and maintained in RPMI-1640 (Gibco) supplemented with 10% fetal bovine serum. DU145 cells expressing ESE3/EHF were generated after transfection with ESE3/EHF expression vector and selection with G418 [15]. Where indicated, PrECs, RWPE-1, ESE3KD-PrECs and ESE3KD-RWPE-1 cells were stimulated with IL-6 50 ng/mL for 2 or 4 h or DMSO as control.

### RNA extraction, quantitative real-time RT-PCR and siRNAs

RNA was extracted by Direct-zol RNA Mini-prep kit (Zymo Research). Quantitative real-time RT-PCR (qRT-PCR) was carried out using 20 ng of RNA as template for SYBR Green Fast One Step kit (Qiagen). qRT-PCR primers are reported in Supplementary Table S1. For transient gene knockdown cells were transfected with siRNAs directed to IL-6 (siRNA Silencer select, Ambion) or control (siGL3) siRNA [17] using jetPRIME (Polyplus).

### Soft agar and *in vitro* prostatosphere forming and self-renewal assay

Soft-agar assays were performed as previously described [32]. The prostatosphere assay was previously described [17]. The sphere forming efficiency (SFE) was determined as percentage of prostatosphere relative to the number of cells plated at the start of the experiment. Each experiment was carried out in triplicate and repeated at least three times.

### Reporter constructs and luciferase assays

To analyze IL-6 activity we used IL-6 responsive luciferase reporter pGL3-basic-IL-6 provided by W. Farrar [33]. Luciferase reporter assays were performed as previously described [17]. Results were normalized to Renilla luciferase and expressed as Relative Luciferase Activity (RLA). Each experiment was performed in triplicate and repeated at least three times.

### Animals and tumor xenografts

Mice were purchased from the Harlan Laboratories. Mice were maintained under pathogen-free conditions with food and water provided ad libitum and their general health status was monitored daily. All protocols involving animals were conducted in conformity with the institutional guidelines for animal experimentation and in compliance with national and international policies. Study protocol was approved by the Swiss Veterinary Authority. For *in vivo* self-renewal experiments prostatosphere-derived ESE3KD-PrECs cells (2 × 10^5^ cells/site) were inoculated with Matrigel in the flank of NOD. Cg-Prkdc^scid^ Il2rg^tm1Wjl^/SzJ (NSG) mice (*n* = 4/ group). Tumor size was monitored twice a week with caliper. Primary tumor xenografts derived from ESE3KD-PrECs prostatospheres were dissociated into single cell suspensions and implanted subcutaneously with Matrigel (2 × 10^5^ cells/site) in NSG mice for two more generations (*n* = 4/group). For systemic treatment with JAK-STAT inhibitor (NVP), mice were injected with a dose of 100 mg/kg of NVP-BSK805 by oral gavage every two days for 4 weeks [34, 35].

### Immunoblot and immunocytochemistry

Immunoblots were carried out using anti-IL-6 Rabbit Polyclonal antibody (NeoBioLab),1:500 in I-block and tubulin as control as previously described (Albino et al., 2016). For immunocytochemistry (ICC), harvested cells were washed in PBS by centrifugation and then the concentration adjusted to 5 × 10^6^ cells/ml in PBS. Cells were attached to slides using Cytospin Cytocentifuge (Thermo Scientific) at 800 rpm for 4 minutes. Cells were fixed and permeabilized with Acetone:Methanol, 1:1. After blocking with 5% BSA cells were incubated with anti-STAT3 total (Cell Signaling #9139) 1:400, and anti-STAT3 TYR705 (Cell Signaling #9145) 1:400, antibodies. Cell nuclei were counterstained with hematoxylin solution and finally, the sections were dehydrated and mounted in a suitable organic mounting medium.

### Flow cytometry

All steps for flow cytometry were performed in PBS supplemented with 0.5% BSA, and 2 mM EDTA. STAT3-TYR705 was purchased from Bioconcept (Anti-human Phospho-Stat3 /D3A7) alexa 647, cat. #4324) and used for analysis in accordance with the manufacturer's instructions. Cell sorting was performed with a FACSAria III sorter (BD Biosciences).

### Chromatin immunoprecipitation

Computational search for ETS binding sites on selected gene promoters was performed using Motifviz (biowulf.bu.edu/MotifViz). Chromatin immunoprecipitation was performed with anti-ESE3 (Clone 5A5.5, Lab Vision, Fremont, CA USA); anti AcH3 (Upstate, Millipore); anti H3K9 2met (Upstate, Millipore); anti H3K27 3met (Upstate, Millipore) and IgG control antibody. Samples were analyzed as previously described [16] by qRT-PCR. Primer sets are reported in Supplementary Table S1.

### Correlation analysis, gene signature, functional annotation and gene set enrichment analysis (GSEA)

The EHRO, Taylor and Grasso [20, 21, 36] human prostate cancer datasets were retrieved from GEO. Only raw intensity data for prostate cancer samples were considered. Data were processed in R using the Bioconductor package “oligo” for Affymetrix arrays: sets were separately RMA (Robust Multi-Array Average) normalized (with background correction) and quantile normalized at the probe level. Log2, normalized expression values for ESE3/EHF and IL-6 genes were extracted and their correlation was tested by Pearson's product moment correlation coefficient. Genes were centered and scaled. Scatter plots of ESE3/EHF vs IL-6 have been drawn and lines of best fit calculated. Correlation analysis was performed in indicated human prostate cancer datasets. Pearson test was used, which estimates a correlation value “r” and a significance p-val (r > 0 < 1, direct correlation; r < 0 > −1, inverse correlation).

To extract the gene signature, expression of genes EHF and IL-6 was dichotomized based on the following threshold (for EHF 25th percentile and for IL-6 75th percentile). Samples with low EHF and high IL-6 (*n* = 51, Ehro et al., 2013) were selected and compared to all the others (*n* = 494, Ehro et al., 2013) by *t-test* and results were retained if FDR (False Discovery Rate) < 0.05. The resulted lists of genes were then divided into activated and repressed genes respectively. Transcription factor analysis was carried using *Enrich*. For ETS occupancy we used published ChIP-Seq data for EHF in CALU3 cells [37] and ERG in VCaP / LNCaP [38]. The ChIP-Seq data was annotated using gencode-v19 human genome database. Functional annotation of the genes significantly deregulated in IL-6_high_/ESE3_low_ was obtained using metacore tools. GSEA software was used to identify groups of functionally related genes. Gene sets with an FDR < 0.05 were considered significant. The gene ranking metric in the weighted enrichment score was SNR (signal-to-noise ratio), and *P* values were calculated using 1000 permutations of the genes.

## SUPPLEMENTARY MATERIALS TABLE


