# Supplementary material for: The ETS factor ESE3/EHF represses IL-6 preventing STAT3 activation and expansion of the prostate cancer stem-like compartment
Source: Oncotarget. 2016 Oct 8;7(47):76756–68. doi: 10.18632/oncotarget.12525 (PMC5363547; doi:10.18632/oncotarget.12525)
Supplement: Supplementary file 1 [file oncotarget-07-76756-s001.pdf]

# The ETS factor ESE3/EHF represses IL-6 preventing STAT3 activation and expansion of the prostate cancer stem–like compartment

## Supplementary Materials

**Supplementary Table S1: Primer sets for qRT-PCR and ChIP experiments**

| Assay | Gene/Region | Sequence 5′ to 3′ | Primer Name |
|---|---|---|---|
| qRT-PCR | *IL6* | ccacacagacagccactcac | IL6 LEFT |
| qRT-PCR | *IL6* | tttcagccatctttggaagg | IL6 RIGHT |
| qRT-PCR | IL6 | agtgaggaacaagccagagc | IL6 set3 LEFT |
| qRT-PCR | IL6 | agatgcaataaccacccctg | IL6 set3 RIGHT |
| qRT-PCR | *Stat3* | ggaggagttgcagcaaaaag | STAT3 LEFT |
| qRT-PCR | *Stat3* | gattctctcctccagcatcg | STAT3 RIGHT |
| qRT-PCR | *Lin28A* | agcatgcagaagcgcagatcaa | Lin28A LEFT |
| qRT-PCR | *Lin28A* | agagcatcagccatatggtagc | Lin28A RIGHT |
| qRT-PCR | *Lin28B* | gccccttggatattccagtc | Lin28B LEFT |
| qRT-PCR | *Lin28B* | cttccaaaggccttgagtca | Lin28B RIGHT |
| qRT-PCR | *BMI-1* | tcatccttctgctgatgctg | BMI-1 LEFT |
| qRT-PCR | *BMI-1* | ccgatccaatctgttctggt | BMI-1 RIGHT |
| qRT-PCR | *NANOG* | cagtctggacactggctgaa | NANOG LEFT |
| qRT-PCR | *NANOG* | ctcgctgattaggctccaac | NANOG RIGHT |
| qRT-PCR | *POU5F1* | agcgatcaagcagcgactat | POU5F1 LEFT |
| qRT-PCR | *POU5F1* | tagcctggggtaccaaaatg | POU5F1 RIGHT |
| qRT-PCR | *β-ACTIN* | attggcaatgagcggttc | β-ACTIN LEFT |
| qRT-PCR | *β-ACTIN* | ggatgccacaggactccat | β-ACTIN RIGHT |

| Assay | Gene/Region | Sequence 5′ to 3′ | Predicted EBS | Primer Name |
|---|---|---|---|---|
| Real Time- ChIP | *IL6 EBS1* | acagctgggaagacgagaaa | –550 –557 | IL6_EBS1 LEFT |
| Real Time- ChIP | *IL6 EBS1* | ggaagttcgtgttcatgataaaa | –550 –557 | IL6_EBS1 RIGHT |
| Real Time- ChIP | *GAPDH* | tcctcctgtttcatccaagc | None | GAPDH_LEFT |
| Real Time- ChIP | *GAPDH* | tagtagccgggccctacttt | None | GAPDH_RIGHT |
